# Supplementary material for: From sequence to enzyme mechanism using multi-label machine learning
Source: BMC Bioinformatics. 2014 May 19;15:150. doi: 10.1186/1471-2105-15-150 (PMC4229970; doi:10.1186/1471-2105-15-150)
Supplement: Additional file 2 — Java code of ml2db. Additional file ml2db_code.tar.gz contains the Java source code to run the multi-label machine learning experiments and save the results to database. The code’s Javadoc is included. [file 1471-2105-15-150-S2.zip › additional file 2/ml2db/ml2db/doc/mulan/evaluation/measure/LabelBasedBipartitionMeasureBase.html]

LabelBasedBipartitionMeasureBase


JavaScript is disabled on your browser.


- Overview
- Package
- Class
- Use
- Tree
- Deprecated
- Index
- Help

- Prev Class
- Next Class

- Frames
- No Frames

- All Classes

- Summary:
- Nested |
- Field |
- Constr |
- Method

- Detail:
- Field |
- Constr |
- Method


mulan.evaluation.measure

## Class LabelBasedBipartitionMeasureBase

- java.lang.Object
- - mulan.evaluation.measure.MeasureBase
  - - mulan.evaluation.measure.BipartitionMeasureBase
    - - mulan.evaluation.measure.LabelBasedBipartitionMeasureBase

- All Implemented Interfaces:
  :   java.io.Serializable, Measure

  Direct Known Subclasses:
  :   LabelBasedFMeasure, LabelBasedPrecision, LabelBasedRecall, LabelBasedSpecificity

  ---

    

  ```
  public abstract class LabelBasedBipartitionMeasureBase
  extends BipartitionMeasureBase
  ```

  Base class for label-based bipartition measures

  Version:
  :   2012.05.29

  Author:
  :   Grigorios Tsoumakas

  See Also:
  :   Serialized Form

- - ### Constructor Summary

    Constructors

    Constructor and Description

    `LabelBasedBipartitionMeasureBase(int aNumOfLabels)`

    Creates a new instance of this class
  - ### Method Summary

    Methods

    | Modifier and Type | Method and Description |
    | --- | --- |
    | `void` | `reset()` Resets the cumulated measure value, so the process of computation can be started from beginning (e.g. |
    | `void` | `updateBipartition(boolean[] bipartition, boolean[] truth)` Updates the measure based on an example |

    - ### Methods inherited from class mulan.evaluation.measure.MeasureBase

      `makeCopy, toString, update`
    - ### Methods inherited from class java.lang.Object

      `equals, getClass, hashCode, notify, notifyAll, wait, wait, wait`
    - ### Methods inherited from interface mulan.evaluation.measure.Measure

      `getIdealValue, getName, getValue`

- - ### Constructor Detail


    - #### LabelBasedBipartitionMeasureBase

      ```
      public LabelBasedBipartitionMeasureBase(int aNumOfLabels)
      ```

      Creates a new instance of this class

      Parameters:
      :   `aNumOfLabels` - the number of labels
  - ### Method Detail


    - #### updateBipartition

      ```
      public void updateBipartition(boolean[] bipartition,
                           boolean[] truth)
      ```

      **Description copied from class: `BipartitionMeasureBase`**

      Updates the measure based on an example

      Parameters:
      :   `bipartition` - the predicted bipartition
      :   `truth` - the ground truth


    - #### reset

      ```
      public void reset()
      ```

      **Description copied from interface: `Measure`**

      Resets the cumulated measure value, so the process of computation can be started
      from beginning (e.g. for a new series of outputs from learning task).


- Overview
- Package
- Class
- Use
- Tree
- Deprecated
- Index
- Help

- Prev Class
- Next Class

- Frames
- No Frames

- All Classes

- Summary:
- Nested |
- Field |
- Constr |
- Method

- Detail:
- Field |
- Constr |
- Method
